# Supplementary material for: Immunogenic Death of Hepatocellular Carcinoma Cells in Mice Expressing Caspase-Resistant ROCK1 Is Not Replicated by ROCK Inhibitors
Source: Cancers (Basel). 2022 Nov 30;14(23):5943. doi: 10.3390/cancers14235943 (PMC9740421; doi:10.3390/cancers14235943)
Supplement: Supplementary file 1 [file cancers-14-05943-s001.zip › cancers-2027533-supplementary v5/cancers-2027533-supplementary.docx]

Supplementary Materials: Immunogenic Death of Hepatocellular Carcinoma Cells in Mice Expressing Caspase-Resistant ROCK1 is not Replicated by ROCK Inhibitors

Gregory Naylor, Linda Julian, Steven Watson-Bryce, Margaret Mullin, Robert J. Nibbs and Michael F. Olson


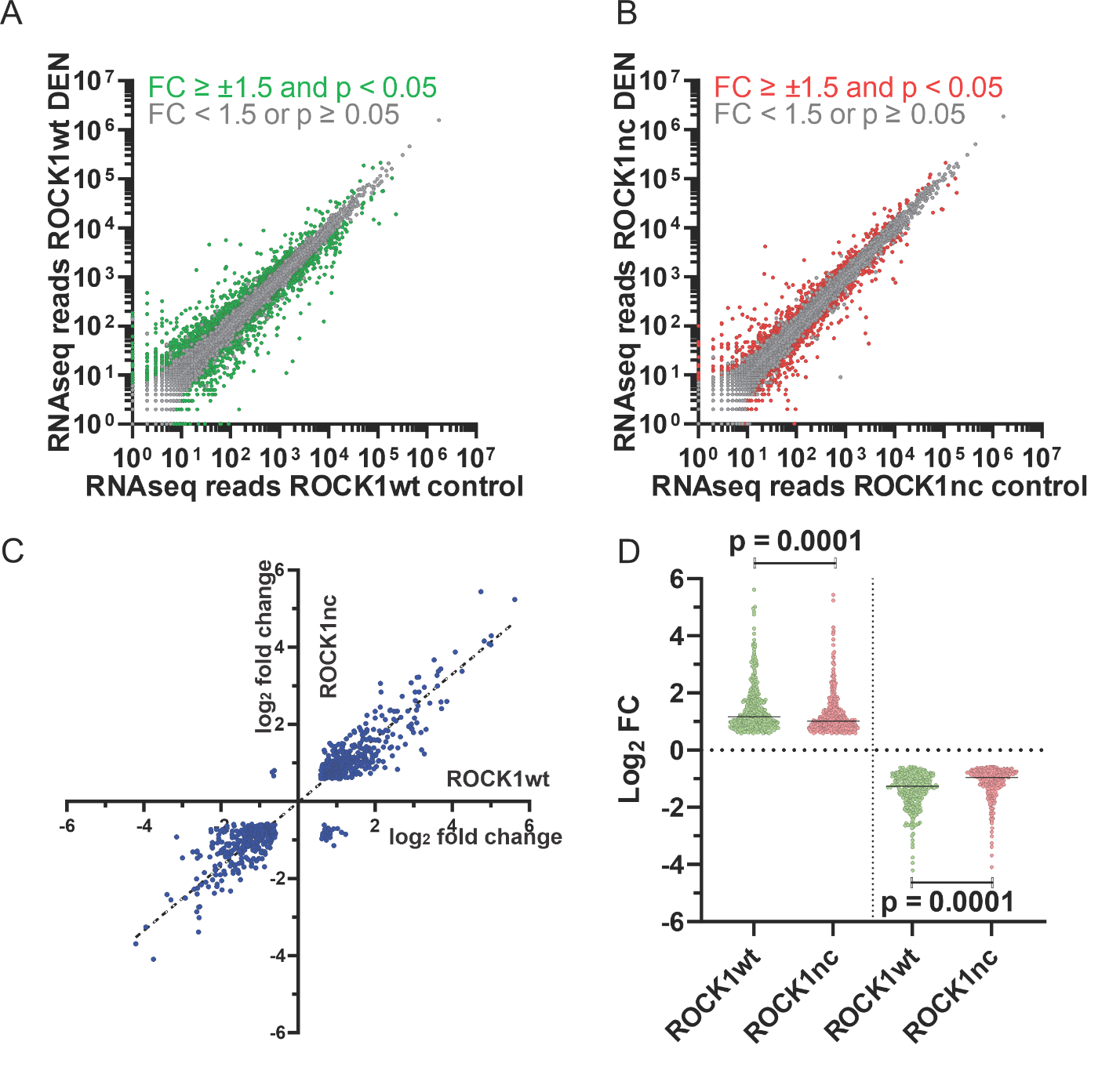


**Figure S1.** RNA sequencing of untreated and DEN-treated ROCK1wt and ROCK1nc livers. **A.** Log_10_-log_10_ scatter plots of mean RNAseq reads for ROCK1wt livers that had been treated for 72 hours with vehicle vs DEN, where p < 0.05 and fold-change (FC) differences > ± 1.5X indicated with green dots, p ≥ 0.05 or FC ≤ ± 1.5X indicated with grey dots. **B.** Log_10_-log_10_ scatter plots of mean RNAseq reads for ROCK1nc livers that had been treated for 72 hours with vehicle vs DEN, where p < 0.05 and fold-change (FC) differences > ± 1.5X indicated with red dots, p ≥ 0.05 or FC ≤ ± 1.5X indicated with grey dots.**C**. Scatter plot of RNAseq log_2_ FC for 72 hour treatment with vehicle versus DEN from ROCK1wt and ROCK1nc mouse livers. Dotted line shows Deming regression line with calculated slope = 0.8313. **D.** RNAseq Log_2_ FC for sequence reads increased in DEN versus vehicle treated ROCK1wt and ROCK1nc mice (left) and for sequence reads decreased in DEN versus vehicle treated ROCK1wt and ROCK1nc mice (right). Horizontal line = median. Unpaired Student’s t-test, p values less than 0.05 indicated in bold typeface.


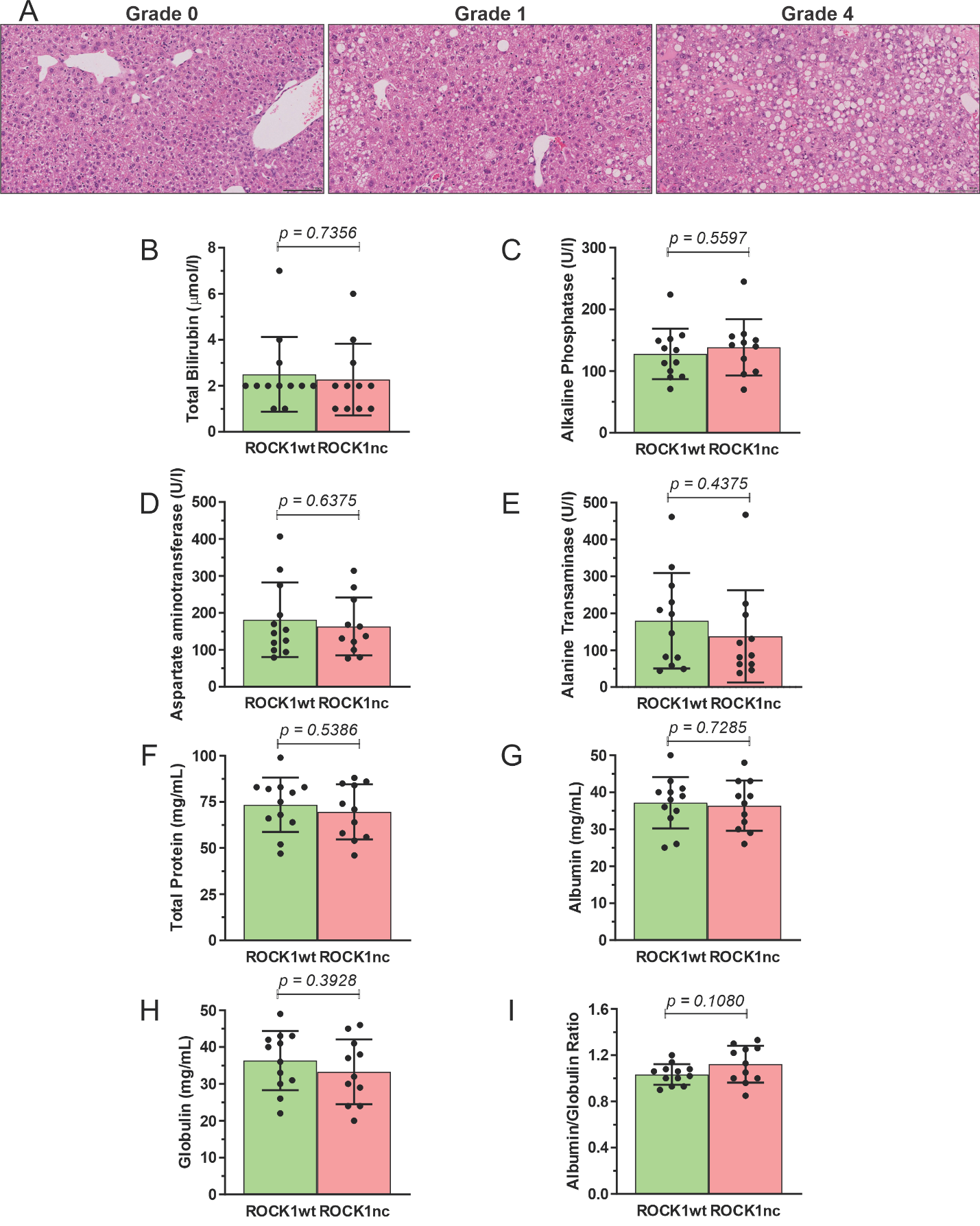


**Figure S2.** Biochemical markers of liver function and health are not different between ROCK1wt and ROCK1nc mice with DEN-induced tumours. **A**. Example liver sections that were evaluated as grade 0, 1 and 4. Scale bar = 100 µm. Serum levels of: **B.** total bilirubin; **C**. alkaline phosphatase; **D**. aspartate aminotransferase; **E**. alanine transaminase; **F**. total protein; **G**. albumin; H. globulins and I. ratio of albumin/globulin were not significantly different between ROCK1wt and ROCK1nc mice with DEN-induced tumours. Means ± standard deviation, ROCK1wt n = 12 mice, ROCK1nc n = 11 mice. Unpaired Student’s t-test.


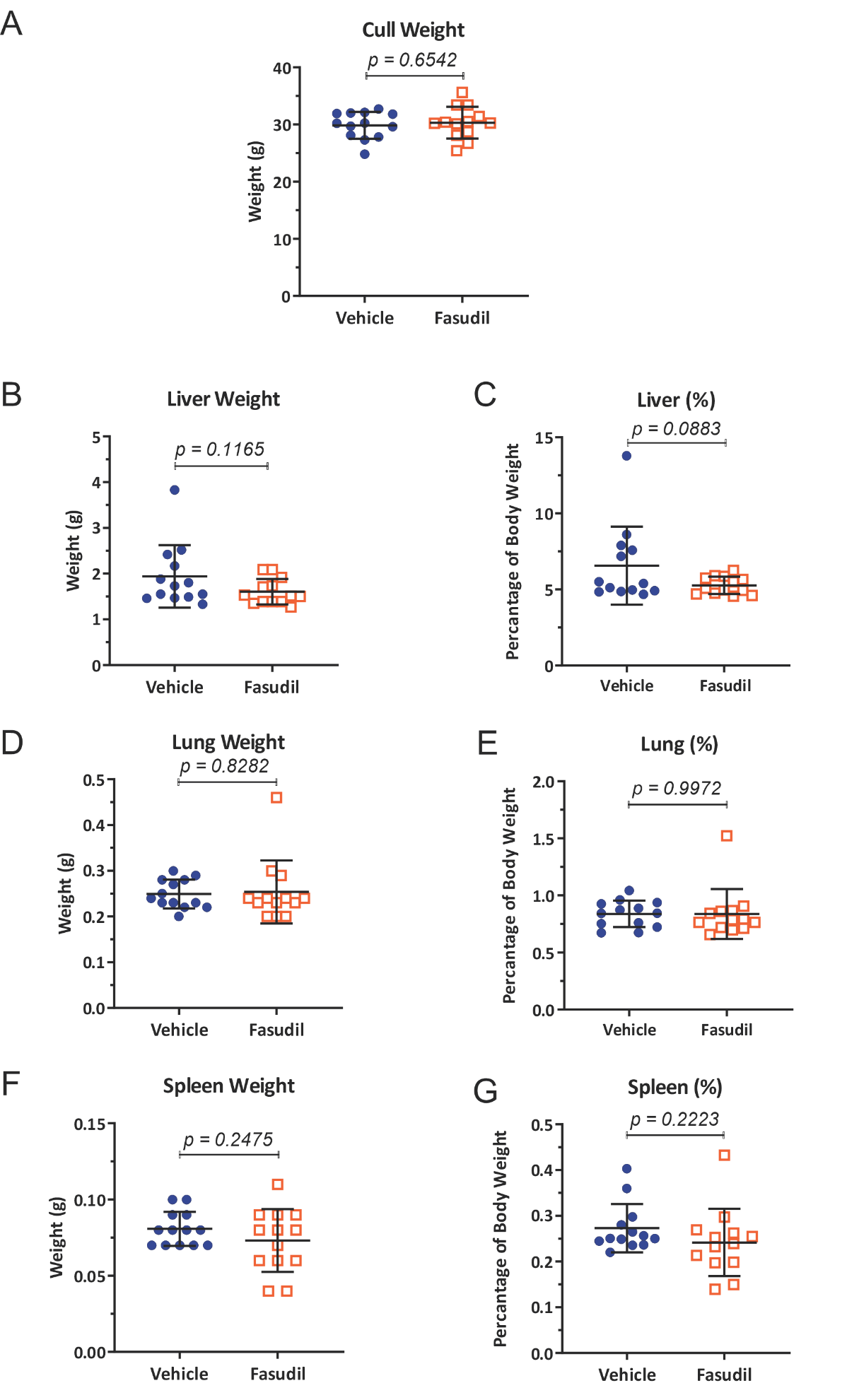


**Figure S3. No differences in body, liver, lung or spleen weights between mice treated with vehicle or fasudil.** Weights at experimental endpoint of: **A.** whole mice; **B.** livers; **C.** livers as a percentage of body weight; **D.** lungs; **E.** lungs as a percentage of body weight; **F.** spleens; **G.** spleens as a percentage of body weight. There were no significant differences between mice treated with vehicle or fasudil. Means ± standard deviation, vehicle n = 13 mice, fasudil n = 13 mice. Unpaired Student’s t-test.


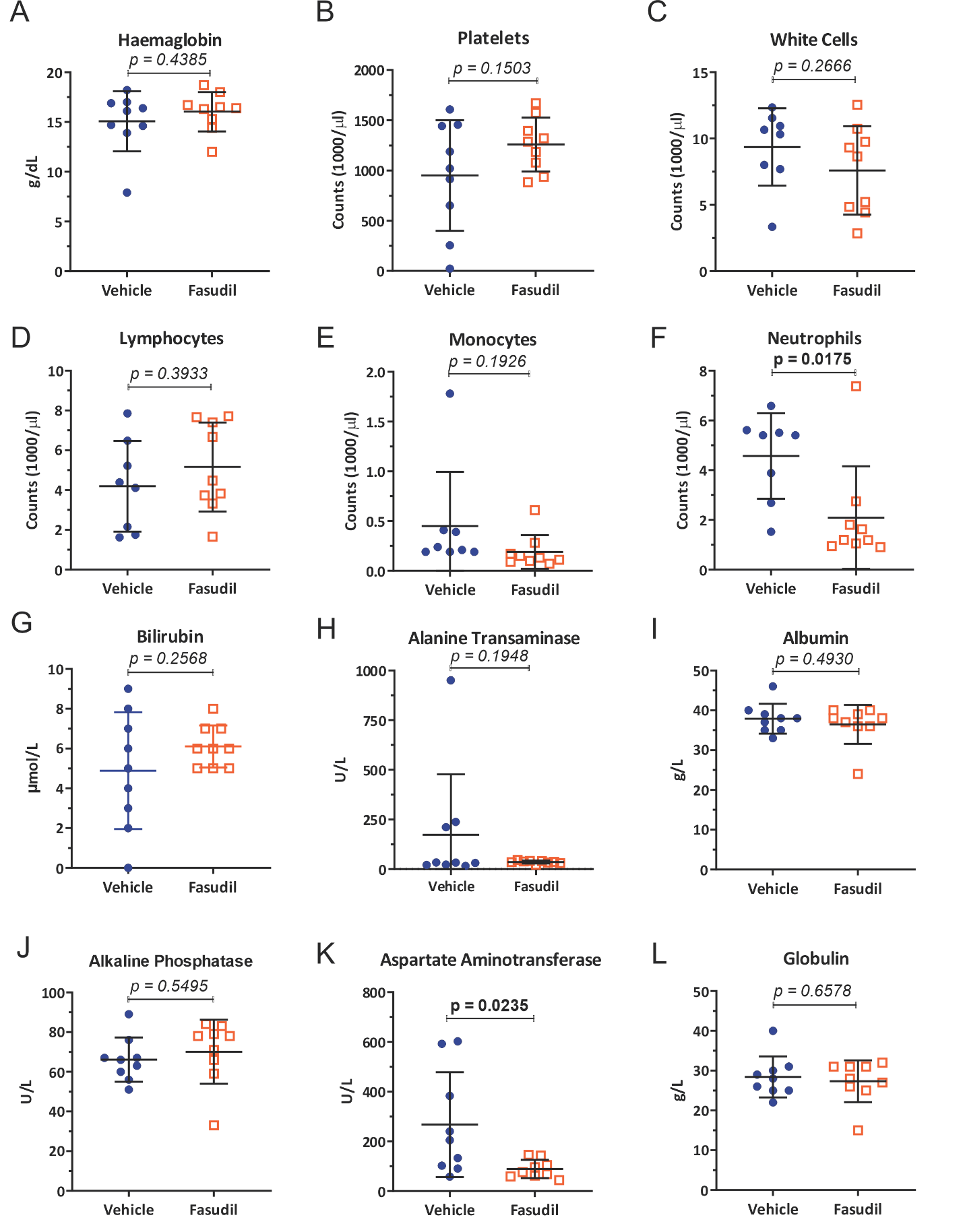


**Figure S4.** Fewer circulating neutrophils and reduced aspartate aminotransferase levels in fasudil treated mice relative to vehicle control. Blood from vehicle or fasudil treated mice was analyzed for: A. Haemoglobin levels; numbers of B. platelets; C. white cells; D. lymphocytes; E. monocytes; F. neutrophils. Serum from vehicle or fasudil treated mice was analyzed for markers of liver function and health: G. bilirubin; H. alanine transaminase; I. albumin; J. alkaline phosphatase; K. aspartate aminotransferase; L. globulins. Means ± standard deviation, vehicle n = 9 mice, fasudil n = 9 mice. Unpaired Student’s t-test, p values less than 0.05 indicated in bold typeface.


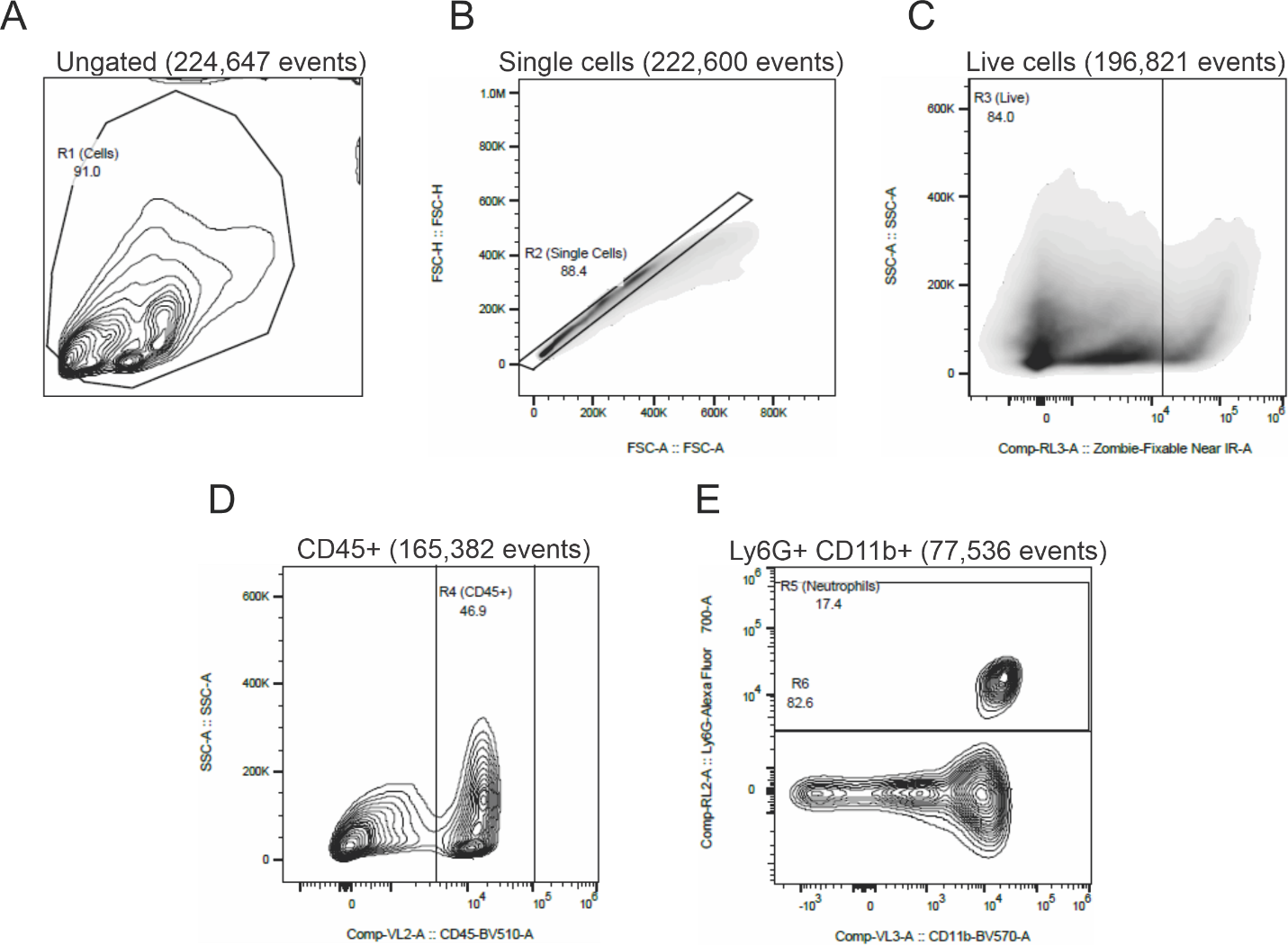


**Figure S5. Representative flow cytometry gating contour plots for immune cell quantification.** Liver cell suspensions were introduced into the flow cytometer and **A.** counted, **B.** gated for single cells, **C**. gated for live cells based on exclusion of Zombie NIR live dead dye, **D.** gated for CD45+ staining and **E.** gated for Ly6G+ CD11b+. In this way, the proportion of neutrophils in samples was determined.


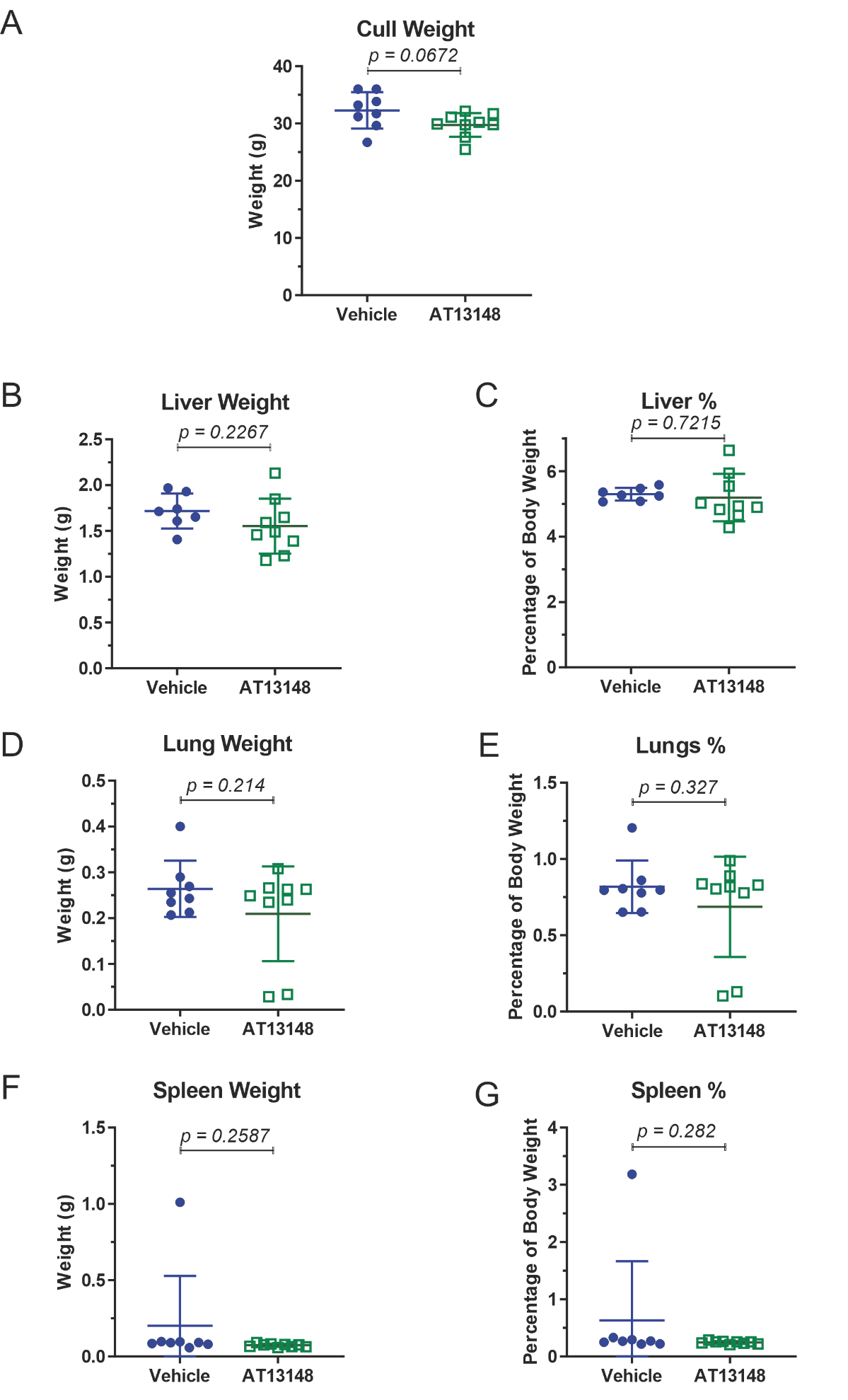


**Figure S6. No differences in body, liver or lung weights between mice treated with vehicle or AT13148.** Weights at experimental endpoint of: **A.** whole mice; **B.** livers; **C.** livers as a percentage of body weight; **D.** lungs; **E.** lungs as a percentage of body weight; **F.** spleens; **G.** spleens as a percentage of body weight. There were no significant differences between mice treated with vehicle or AT13148, with the exception of spleen weights that were significantly smaller when comparing weights, but not when compared as percentages of body weights. Means ± standard deviation, vehicle n = 9 mice, AT13148 n = 9 mice. Unpaired Student’s t-test.


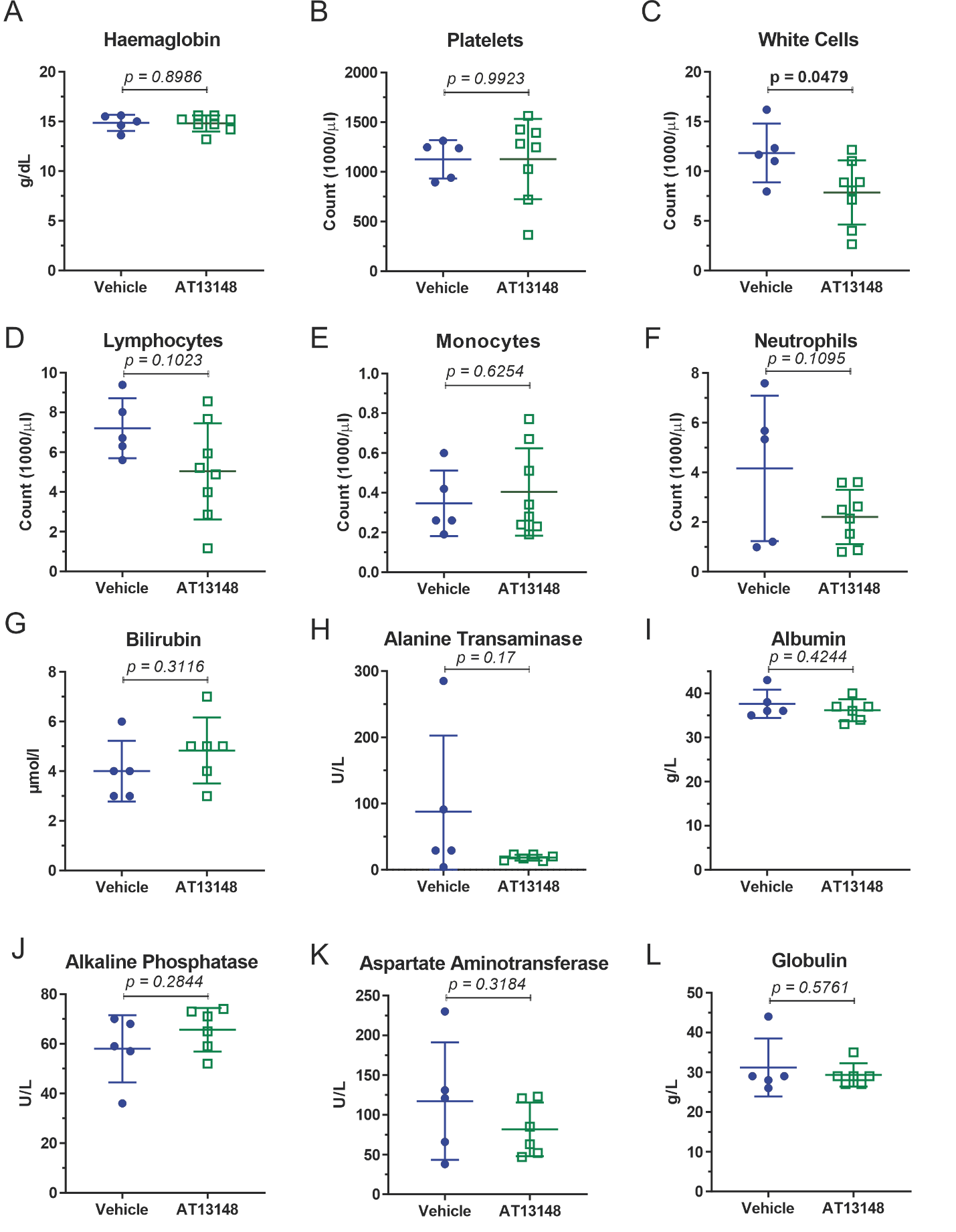


**Figure S7.** No significant differences in circulating blood cells or biochemical markers of liver health and function between vehicle or AT13148 treated mice. Blood from vehicle or AT13148 treated mice was analyzed for: A. haemoglobin levels; numbers of B. platelets; C. white cells; D. lymphocytes; E. monocytes; F. neutrophils. Serum from vehicle or AT13148 treated mice was analyzed for markers of liver function and health: G. bilirubin; H. alanine transaminase; I. albumin; J. alkaline phosphatase; K. aspartate aminotransferase; L. globulins. Means ± standard deviation, vehicle n = 5 mice, AT13148 n = 8 mice. Unpaired Student’s t-test, p values less than 0.05 indicated in bold typeface.


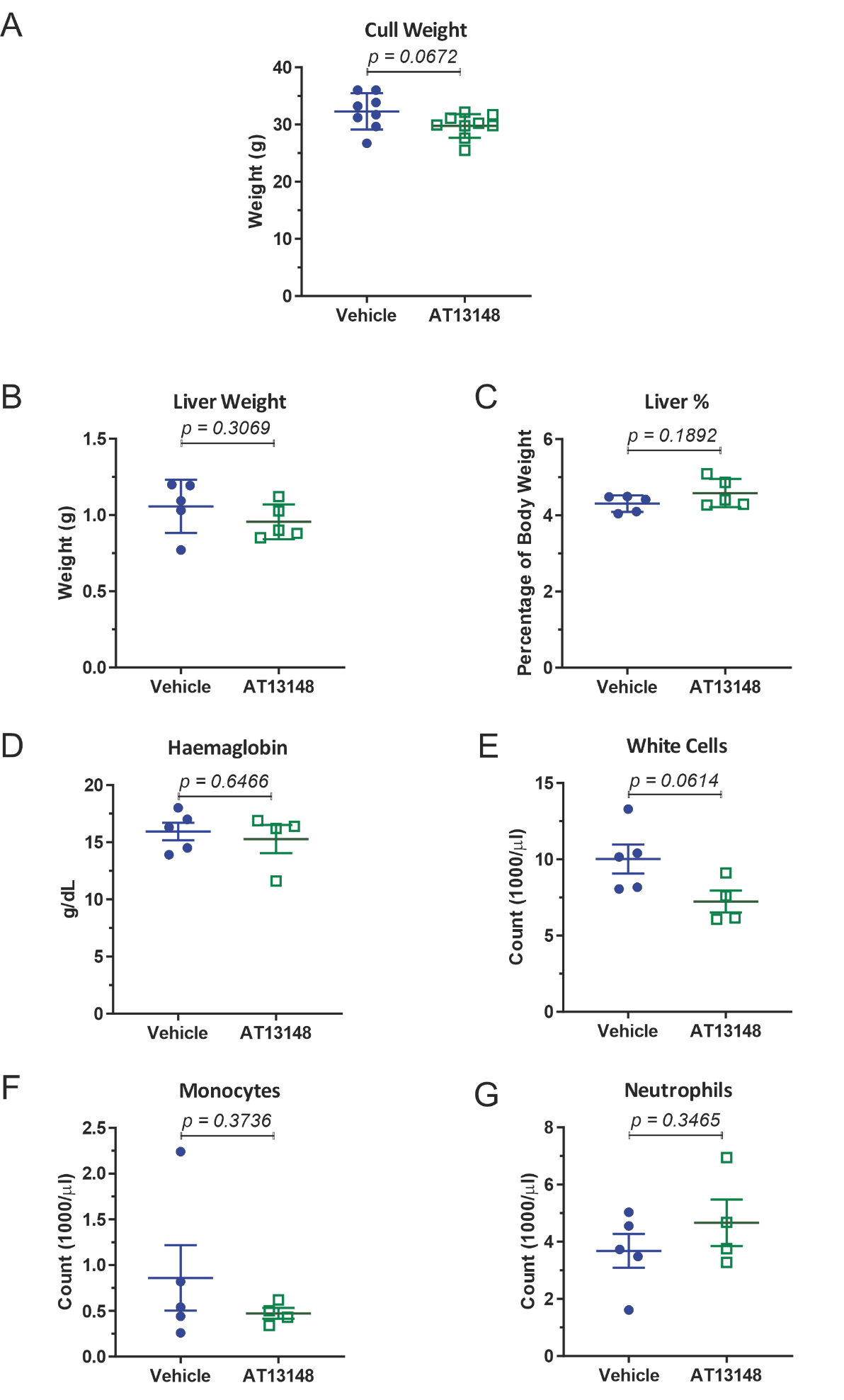


**Figure S8.** No significant differences in body or liver weights, blood haemoglobin levels, or circulating white cells, monocytes or neutrophils between vehicle or AT13148 treated mice following acute DEN treatment. Weights at experimental endpoint of: A. whole mice; B. livers; C. livers as a percentage of body weight. Blood from vehicle or AT13148 treated mice was analyzed for: D. haemoglobin levels; numbers of E. white cells; F. monocytes; G. neutrophils. Means ± standard deviation, vehicle n = 5 mice, AT13148 n = 4 mice. Unpaired Student’s t-test.


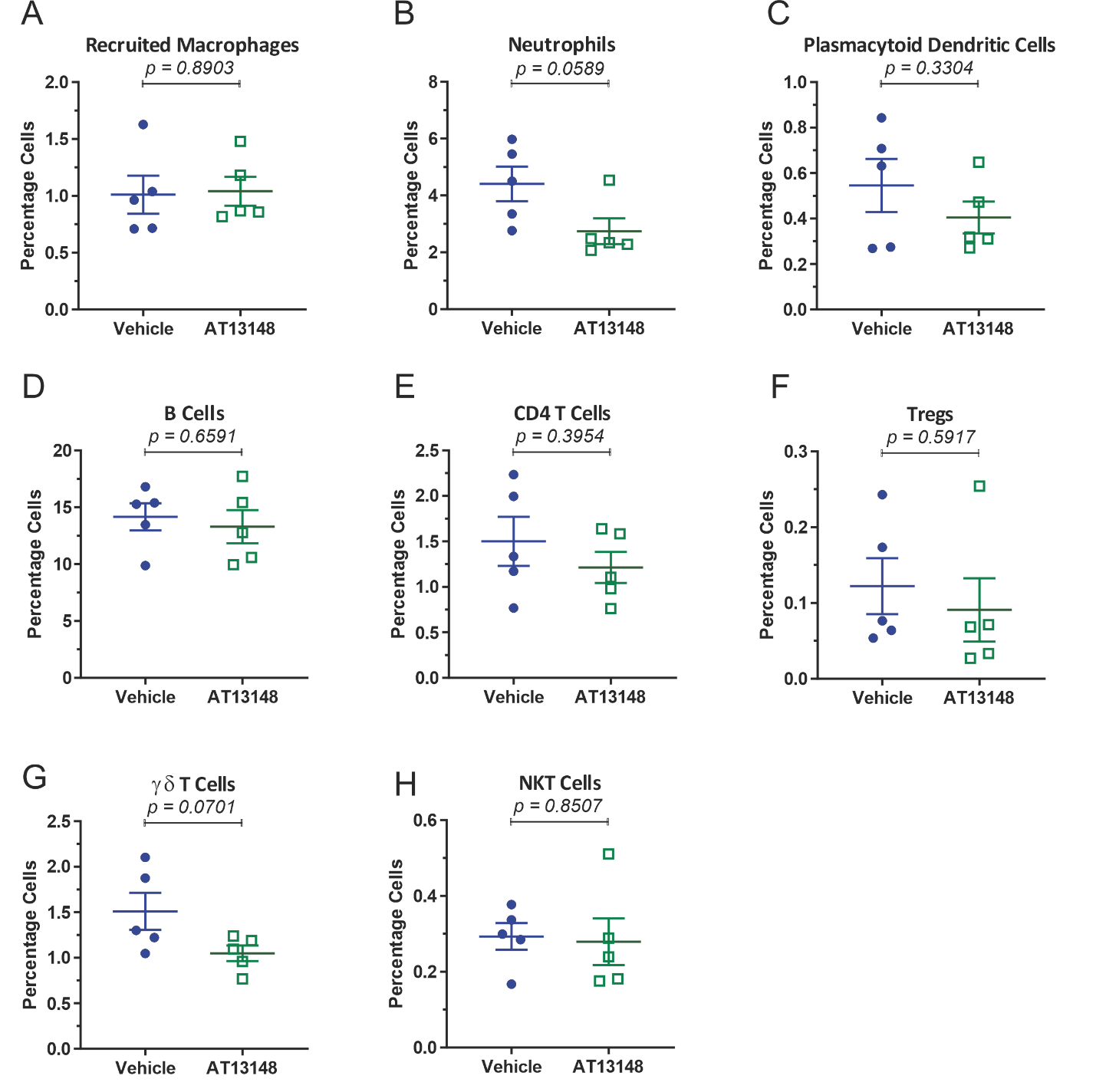


**Figure S9. Immune cell recruitment to livers following acute DEN treatment in vehicle or AT13148 treated mice.** Liver cell suspensions were analyzed by flow cytometry to determine the proportions of immune cells relative to total cell numbers counted of: **A.** recruited macrophages; **B.** neutrophils; **C.** plasmacytoid dendritic cells; **D.** B cells; **E.** CD4^+^ T cells; **F.** Tregs; **G.** γδ T cells; **H.** NKT cells. Means ± standard deviation, vehicle n = 5 mice, AT13148 n = 5 mice. Unpaired Student’s t-test.

**Video 1. Time lapse of apoptotic ROCKwt hepatocytes.** ROCK1wt hepatocytes were plated on 6-well glass bottom dishes that had been coated with collagen. Cells were serum-starved overnight prior to induction of apoptosis with 1 µM ABT-199 diluted in serum-free starvation medium. Immediately after induction of apoptosis, the dishes were transferred to the microscope and brightfield time-lapse microscopy images were acquired at 3 minute intervals for up to 120 minutes with a 10X DIC objective using a Nikon TE 2000 microscope with a heated stage and 5% CO2 gas line.

**Video 2. Time lapse of apoptotic ROCKnc hepatocytes.** ROCK1nc hepatocytes were plated on 6-well glass bottom dishes that had been coated with collagen. Cells were serum-starved overnight prior to induction of apoptosis with 1 µM ABT-199 diluted in serum-free starvation medium. Immediately after induction of apoptosis, the dishes were transferred to the microscope and brightfield time-lapse microscopy images were acquired at 3 minute intervals for up to 120 minutes with a 10X DIC objective using a Nikon TE 2000 microscope with a heated stage and 5% CO2 gas line.
